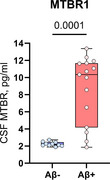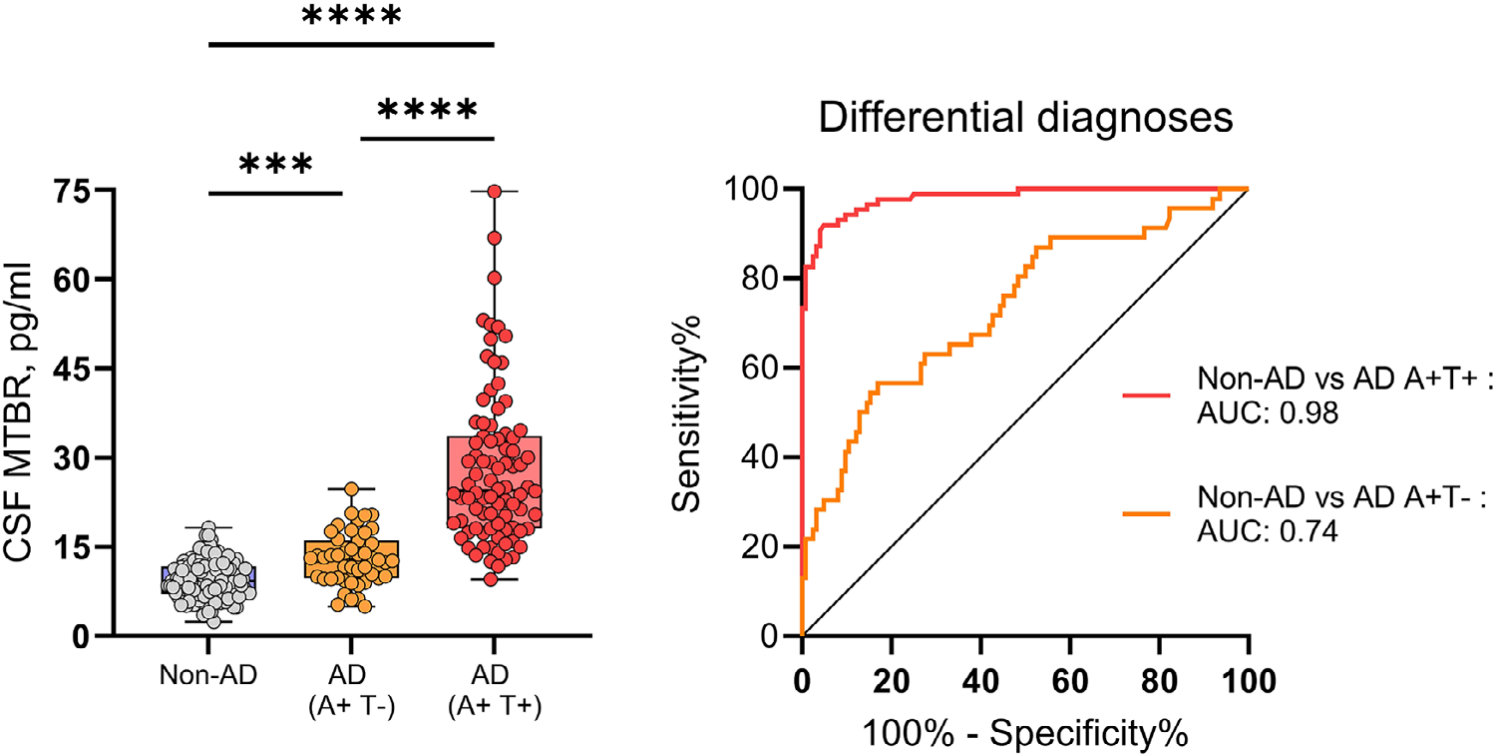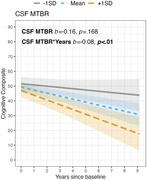# Novel MTBR‐specific immunoassays MTBR‐1 and MTBR‐pTau262 for assessment of tau pathology in Alzheimer's disease

**DOI:** 10.1002/alz70861_108526

**Published:** 2025-12-23

**Authors:** Fernando Gonzalez‐Ortiz, Michael K Turton, Johan Gobom, Gunnar Brinkmalm, Peter Harrison, Anja Hviid Simonsen, Bjørn‐Eivind Kirsebom, Tormod Fladby, Kaj Blennow

**Affiliations:** ^1^ University of Gothenburg, Department of Psychiatry And Neurochemistry, Institute of Neuroscience And Physiology, The Sahlgrenska Academy At The University Of Gothenburg, Mölndal Sweden; ^2^ Bioventix Plc, Farnham, Gothenburg Sweden; ^3^ Sahlgrenska University Hospital, Mölndal Sweden; ^4^ Department of Psychiatry and Neurochemistry, Institute of Neuroscience and Physiology, the Sahlgrenska Academy at the University of Gothenburg, Mölndal, Gothenburg Sweden; ^5^ Bioventix Plc, Farnham, UK UK; ^6^ Danish Dementia Research Centre, Dept. of Neurology, Copenhagen University Hospital ‐ Rigshospitalet, Copenhagen, Denmark Denmark; ^7^ University Hospital of Northern Norway, Tromsø, Troms Norway; ^8^ Akershus University Hospital, Nordbyhagen, Norway Norway; ^9^ Clinical Neurochemistry Laboratory, Sahlgrenska University Hospital, Mölndal, Västra Götalands län Sweden

## Abstract

**Background:**

Tau aggregation and neurofibrillary tangle formation are pathological hallmarks of Alzheimer’s disease (AD). Central to tau aggregation is the microtubule‐binding region (MTBR), a critical domain involved in stabilizing microtubules under physiological conditions and driving pathological aggregation of tau in AD. Prior studies have demonstrated that the early R1 segment of the MTBR (encompassing residues 243–254) is significantly enriched in brains with advanced AD pathology, whereas the late R1 segment (residues 260–267) shows no such enrichment. In this study, we present results from a cerebrospinal fluid (CSF) immunoassay (MTBR1) targeting the 225–255 region of tau. Additionally, results on phosphorylated tau at serine 262 (MTBR pTau262) will be presented at AAIC 2025.

**Method:**

We developed and validated a new panel of sheep monoclonal antibodies targeting the early R1 region of the MTBR domain, along with a companion antibody directed at the adjacent pre‐R1 sequence. Antibody specificity was confirmed using immunoprecipitation and mass spectrometry. These antibodies were incorporated into a Simoa‐based immunoassay for CSF and evaluated across three independent cohorts (N = 996).

**Result:**

CSF MTBR1 was able to differentiate between A‐ and A+ individuals in the cohort‐1 (Figure 1, *p* <0.001). In the memory clinic cohort, CSF MTBR1 showed significant increases in A+T‐ individuals compared to control but showed the largest increases in A+T+ (Figure 2). In cohort‐3, CSF MTBR1 was associated with future cognitive decline among A+ individuals supporting its role as a symptomatic marker.

**Conclusion:**

The MTBR1 assay represents the first high‐performing immunoassay capable of detecting MTBR tau fragments in CSF, showing significant elevation in relation to both cognitive impairment severity and tau accumulation. In addition, MTBR‐pTau262 emerges as a promising novel biomarker that reflects MTBR tau species in both CSF and plasma. These markers may serve as a valuable complement to existing plasma biomarkers, potentially improving accessibility and specificity in AD diagnostics and disease monitoring.